# Discovering subnetworks in SBML models

**DOI:** 10.1093/bioinformatics/btaf482

**Published:** 2025-09-04

**Authors:** Joseph L Hellerstein, Lucian P Smith, Lillian T Tatka, Steven S Andrews, Michael A Kochen, Herbert M Sauro

**Affiliations:** eScience Institute, University of Washington, Seattle, WA, 98195, United States; Paul G. Allen School of Computer Science, University of Washington, Seattle, WA, 98195, United States; Department of Bioengineering, University of Washington, Seattle, WA, 98195, United States; Paul G. Allen School of Computer Science, University of Washington, Seattle, WA, 98195, United States; Talus Bioscience, Seattle, WA, 98122, United States; Department of Bioengineering, University of Washington, Seattle, WA, 98195, United States; Software Applications and Technology, Rocky Mountain Division, Applied Research Associates, Inc, Bentonville, AR, 72212, United States; eScience Institute, University of Washington, Seattle, WA, 98195, United States; Department of Bioengineering, University of Washington, Seattle, WA, 98195, United States

## Abstract

**Motivation:**

Many advances in biomedical research are driven by structural analysis, which investigates interconnections between elements in biological systems (e.g. structural analysis of proteins to infer their function). Herein, we consider subnet discovery in chemical reaction networks (CRNs)–discovering a subset of a target CRN, i.e. structurally identical to a reference CRN. Structural analysis techniques such as motif finding and graph mining look for small, arbitrary, and commonly occurring substructures (e.g. three gene feedforward loops). In contrast, subnet discovery looks for larger, specific, and infrequently occurring substructures (e.g. 10 reactions mitogen-activated protein kinase (MAPK) pathway).

**Results:**

We introduce pySubnetSB, an open source Python package for discovering subnets in CRNs that are represented in the Systems Biology Markup Language (SBML) community standard. We show that pySubnetSB achieves large reductions in computational complexity for subnet discovery. For example, in studies of randomly selected target networks with 100 reactions each with a random reference network with 20 reactions, computations are reduced from an infeasible $10^{78}$ evaluations to a more practical $10^{8}$ evaluations. We develop a methodology for assessing the statistical significance of subnet discovery. Last, we study subnets in BioModels for approximately 200 000 pairs of reference and target models. We show that for a reference MAPK pathway, subnet discovery correctly indicates the presence of MAPK function in several target models. The studies also suggest two interesting hypotheses: (a) the potential presence of hidden oscillators in several models in BioModels, and (b) the possibility of a conserved mechanism for intracellular immune response.

**Availability and implenetation:**

pySubnetSB is installed using pip install pySubnetSB, and is hosted at https://github.com/ModelEngineering/pySubnetSB/.

## 1 Introduction

Many insights in biomedical research are driven by structural analysis, which investigates the interconnections between elements in biological systems. Examples include: structural analysis of proteins to infer their function (e.g. Osadchy and Kolodny 2011); analysing chemical pathways to identify drug targets (e.g. Paul *et al.* 2021); structures in chemical reaction networks (CRNs) related to functions such as signal amplification and error detection (Hartwell *et al.* 1999); and the function of gene motifs in gene regulatory networks (e.g. Alon 2007). Structural analysis is often successful because: (a) structural information is much easier to obtain and generally more accurate than dynamics such as species concentrations and reaction fluxes; and (b) biological structures can be closely related to their function (e.g. Sharan and Ideker 2006). Many tools have been developed for analysing biological structures, such as: Basic Local Assignment Search Tool (BLAST) for sequences (McGinnis and Madden 2004); graph mining to discover frequently occurring substructures (Lambusch *et al.* 2018); and developing signatures for substructures related to their biological function (Shellman *et al.* 2013).

Our focus is on CRNs in biological systems. In particular, we develop a tool for discovering subnetworks (hereafter just subnets) in CRNs. By subnet discovery, we mean determining if a reference CRN is structurally identical to a subnet of a target CRN in that there is an equivalence between the reference reactions and chemical species and the reactions and species in a subnet of the target.

At first glance, subnet discovery seems to overlap with motif finding and graph mining, techniques that look for small, arbitrary, and commonly occurring substructures (e.g. three gene feedforward loops (Alon 2007)). In contrast, subnet discovery looks for larger, more specific, and infrequently occurring substructures. For example, Section 4 discusses subnet discovery using the mitogen-activated protein kinase (MAPK) pathway, a specific 10 reactions system that occurs only once in each of its targets.

We believe that subnet discovery is of particular interest for the following use cases.

- Use case 1: Infer function in the target from the reference. If a reference CRN is a subnet of a target CRN, then the functions provided by the reference (e.g. oscillation) might be present in the target.
- Use case 2: Infer conserved mechanisms in targets that have the same reference. If many target CRNs have the same reference as a subnet, then the reference *might* implement mechanisms that are conserved in the targets.

Section 4 provides examples of both use cases. We emphasize that like motif finding and graph mining, the objective of subnet discovery is to identify interesting hypotheses. In general, additional work is required beyond the discovery of the subnet to obtain a research result.

**Figure 1.**
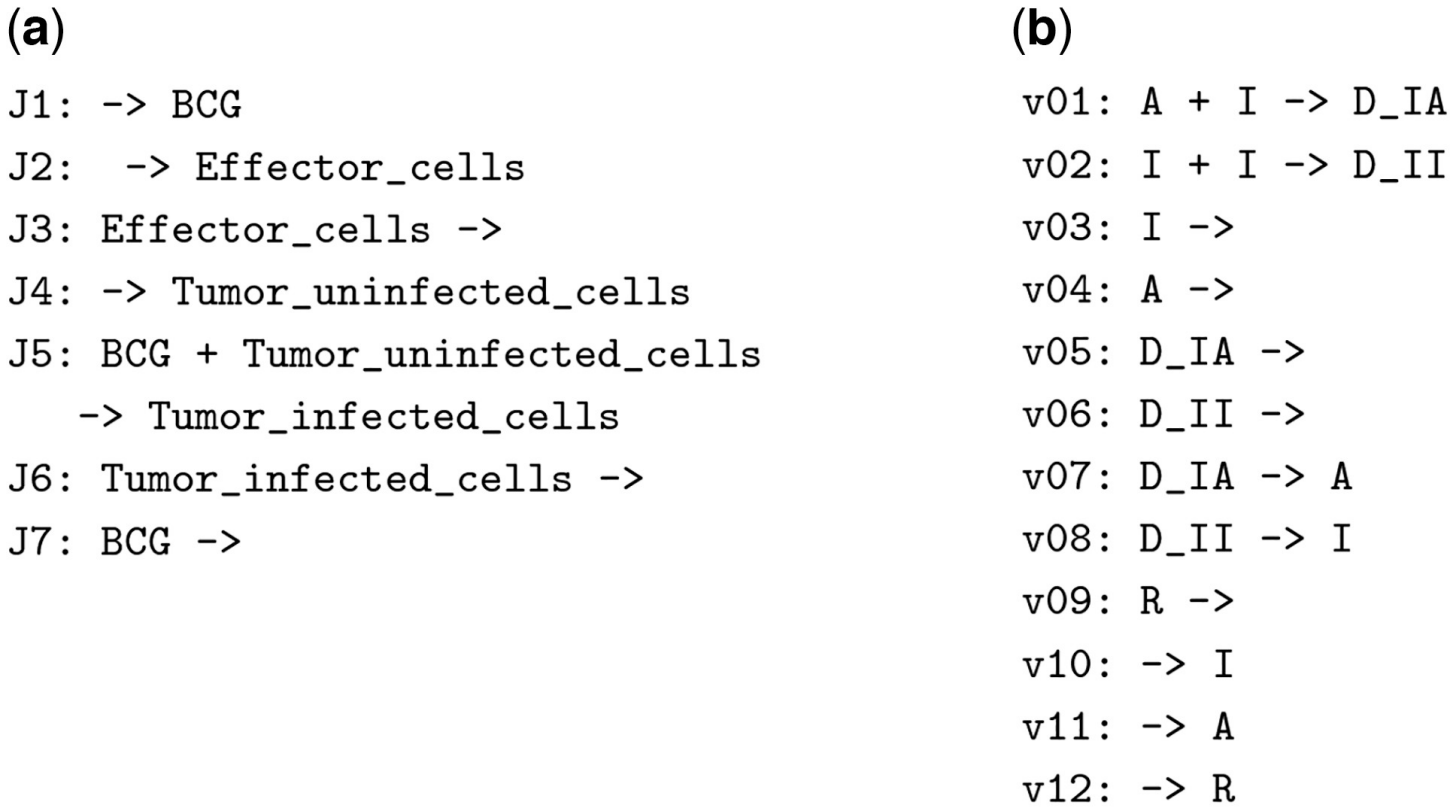
Two models in BioModels. The running example uses Model 1034 (a) as the reference network and Model 351 (b) as the target network.

To illustrate subnet discovery, consider Fig. 1 which displays two models from the curated BioModels repository (Li *et al.* 2010). Model 1034 simulates interactions between tumors and the immune system in Bacillus Calmette–Guerin immunotherapy. Model 351 addresses auxin signaling for position information during plant development. We use Model 1034 as the reference CRN to query Model 351, the target CRN, to discover if there is a subnet of 351, i.e. structurally identical to 1034.

**Figure 2.**
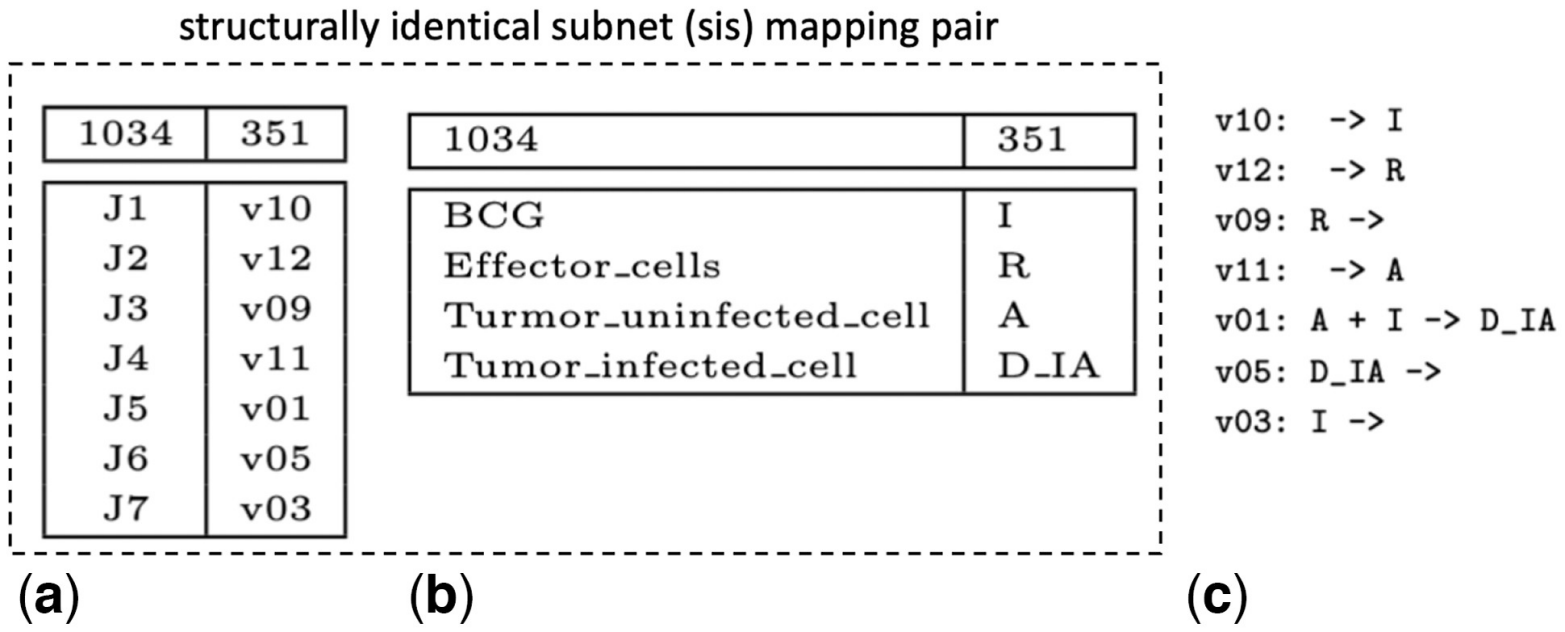
Mapping pairs and inferred subnet. The reaction mapping in (a) in combination with the species mapping in (b) is a mapping pair that identifies the inferred subnet of Model 351 in (c) that is structurally identical to Model 1034.

Although these CRNs address very different biological functions, it turns out that Model 1034 is structurally identical to a subnet of Model 351. This subnet is specified by: (a) a **species mapping** (as in Fig. 2b) that associates each species in 1034 with a species in 351; and (b) a **reaction mapping** (as in Fig. 2a) that associates each reaction in 1034 with a reaction in 351. A **mapping pair** consists of a species mapping and a reaction mapping. The mapping pair in Fig. 2 specifies an **inferred subnet** of Model 351, as displayed in Fig. 2c. This is a **structurally identical subnet (sis) mapping pair** because the inferred subnet is identical to Model 1034 if we change the names of species and reactions as specified by the mapping pair. We note that most mapping pairs do not specify an inferred subnet (e.g. if J1 in 1034 is mapped to v02 in 351), and clearly not every inferred subnet is structurally identical to the reference CRN.

The foregoing is an example of **strong identity**. Strong identity considers both the stoichiometry of reactants in the **reactant stoichiometry matrix** and the stoichiometry of products in the **product stoichiometry matrix**. To illustrate, consider a CRN consisting of a single reaction 2A→A+B. The product and reactant stoichiometry matrices are: $\left(\begin{array}{c} 1\\ 1 \end{array}\right),\left(\begin{array}{c} 2\\ 0 \end{array}\right),$ respectively. The **standard stoichiometry** matrix is the difference between product and reactant stoichiometry matrices, which in our example is $\left(\begin{array}{c} -1\\ 1 \end{array}\right)$. Strong identity means that if we appropriately rename target species and reactions, there is a sub-matrix of the target reactant stoichiometry matrix, i.e. identical to the reference reactant stoichiometry matrix. And it has a similar meaning for the product stoichiometry matrix.

There is also **weak identity** that looks for a subnet in the target with the capability for the same behavior as the reference. By capable of the same behavior, we mean that by adjusting the rate laws of the subnet of the target, we can get the same behavior as the reference. This property holds if the reference and subnet of the target have the same standard stoichiometry matrix since if we analyse a CRN as a system of ordinary differential equations, its behavior depends only on the standard stoichiometry matrix (e.g. Sauro 2014).

Discovering subnets in CRNs is an instance of the subgraph isomorphism problem in graph theory. It is well known that this problem is NP-hard (Hartmanis 1982). To explain this point, consider weak identity, which only considers the standard stoichiometry matrix. A mapping pair for the target CRN specifies a sub-matrix of the target standard stoichiometry matrix that has the same shape as the reference CRN along with permutations of target matrix rows (species) and columns (reactions). If this sub-matrix of the target is identical to the reference stoichiometry matrix, then we have detected weak identity.

In the worst case, we must make matrix comparisons for *all* mapping pairs, which is the computational complexity of a naive approach to subnet discovery. Let $M^{⋆}$ denote the number of possible mapping pairs. Let the superscripts *R*, *T* denote the reference and target CRNs, respectively; let the subscripts *r*, *s* indicate reactions and species; and let *M* denote a count. For example, $M_{r}^{R}$ is the number of reactions in the reference CRN. Then,


(1) $$M^{⋆}=\left(\begin{array}{c} M_{r}^{T}\\ M_{r}^{R} \end{array}\right)M_{r}^{R}!\left(\begin{array}{c} M_{s}^{T}\\ M_{s}^{R} \end{array}\right)M_{s}^{R}!$$

The magnitude of $M^{⋆}$ can be substantial. Consider a modest size reference CRN with 20 reactions and 20 species and a somewhat larger target CRN with 100 reactions and 100 species. Here, $M^{⋆}\approx 10^{78}$. This is only a factor of 100 smaller than $10^{80}$, the current estimate of the number of atoms in the universe.

There are well more than 100 subgraph algorithms (e.g. Conte *et al.* 2004, and the references therein) as well as tens of algorithms for subgraph discovery in biology (e.g. Milano *et al.* 2022, and the references therein). The biology algorithms largely focus on protein–protein interactions and gene regulatory networks, not CRNs.

Special considerations are required to find subgraphs in CRNs. To elaborate, note that the vast majority of existing subgraph algorithms (e.g. McCreesh *et al.* 2020, Cordella *et al.* 2004) use *simple* graphs, graphs whose arcs have a single source and a single destination. But simple graphs do not preserve the semantics of a reaction in subgraph problems. To see this, consider a reference CRN that consists of the single reaction R:A→C, and a target CRN with the single reaction $R^{\prime }:A^{\prime }+B^{\prime }\to C^{\prime }$. Clearly, the reference is not a subnet of the target since both networks have only one reaction, and these reactions have a different number of reactants. However, these differences go undetected with a representation as simple graphs. A simple graph represents the reference network using the arcs: A={(A,R),(R,C)}, and the target with the arcs $A^{\prime }=\{(A^{\prime },R^{\prime }),(B^{\prime },R^{\prime }),(R^{\prime },C^{\prime })\}$. Using the reaction and species mappings that pair the non-primed and primed symbols (e.g. *R* with $R^{\prime }$), we incorrectly infer $R^{\prime }$ includes *R* because $A\subset A^{\prime }$. This is because *representing CRNs as simple graphs often finds subnets in the target that have a different structure from the reference*.

**Figure 3.**
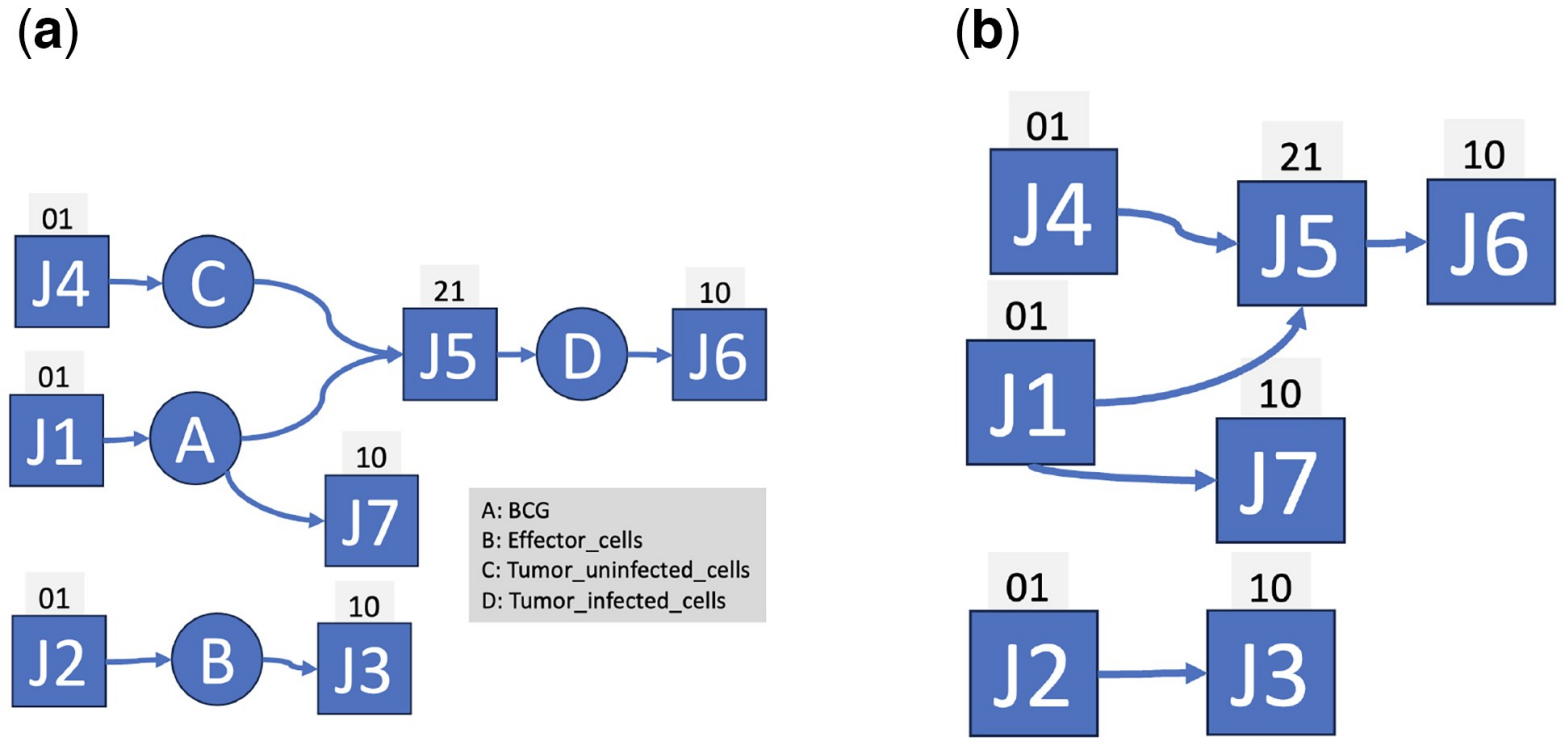
Bipartite graph (a) and reaction monopartite (b) graphs for Model 1034. Squares indicate reactions, and circles indicate species. Text above squares indicate the reaction type (e.g. 01 is 0 reactant, 1 product). The reaction monopartite graph is inferred from the bipartite graph.

This problem can be avoided by representing CRNs as hypergraphs (e.g. Yang *et al.* 2024). By so doing, reactions have two hyperarcs: (a) an inbound arc with a source at each reactant and a single destination at the reaction, and (b) an outbound arc whose single source is the reaction and whose destinations are each product species. For example, the bipartite graph in Fig. 3a has an inbound hyperarc for reaction J5 with a source at its two reactants. The hypergraph representation avoids the problem described above because a target reaction is compatible with a reference reaction only if both reactions have the same number of reactants and products. Almost all subgraph algorithms on hypergraphs find *similar* subgraphs, not subgraph isomorphisms. Similarity algorithms score reaction pairs in many ways such as counting types of subgraphs (e.g. feedforward loops) and commonalities in their participants (e.g. six carbon sugars) (Sharan and Ideker 2006, Zhang *et al.* 2021, Milano *et al.* 2022). As a result, similarity algorithms often require species annotations, and they do not analyse the full topology of connections between species and reactions.

To our knowledge, only Yang *et al.* (2024) proposed an algorithm for finding subgraph isomorphisms in hypergraphs. Even better, this work discusses process-based parallelism (i.e. running on multiple processors) to speed up the computationally intensive processing of the subgraph problem. The authors employ a **constraint-based approach**, such as only considering the mapping of a reference node to a target with at least as many incoming and outgoing arcs as the reference. However, Yang *et al.* (2024) is a general purpose algorithm, not an algorithm specifically targeted at CRNs. For example, the constraint they impose on the number of arcs is useless for reactions in CRNs since all reactions have a single incoming arc and a single outgoing arc. Further, they use the same criteria for all nodes, but we need to use different criteria for reaction and species nodes. Beyond this, Yang *et al.* (2024) does not attempt computational speedup using vectorization (i.e. speedups achieved by using matrix representations instead of graph representations). Vectorization speedups can be substantial. For example, Section 3.3 shows a speed increase of a factor of 40 by using vectorization.

This article introduces pySubnetSB, an open source Python package that discovers subnets in CRNs represented using the community standard of systems biology markup language (SBML) (Hucka *et al.* 2003). pySubnetSB employs a constraint-based approach that greatly reduces computations. In our studies of randomly selected target networks with 100 reaction that each embed a random reference networks with 20 reactions, the number of mapping pairs is reduced from a computationally infeasible $10^{78}$ to a more practical $10^{8}$. Furthermore, our implementation provides large speedups as a result of vectorization and process-based parallelism (e.g. a speedup of 400 in our studies). In addition, we develop a methodology that assesses the statistical significance of subnet discovery. Last, we use pySubnetSB to study subnets in BioModels for approximately 200 000 pairs of reference and target models. We show that for a reference MAPK pathway, subnet discovery correctly indicates the presence of MAPK function in several target models. The studies also suggest a couple of interesting hypotheses: (a) the potential presence of hidden oscillators in several models in BioModels, and (b) the possibility of a conserved mechanism for intracellular immune response.

## 2 Materials and methods

pySubnetSB draws on previous work on constraints-based approaches to subgraph problems, and leverages an analysis of the distribution of reactions in BioModels.

### 2.1 Constraint-based subnet discovery

**Figure 4.**
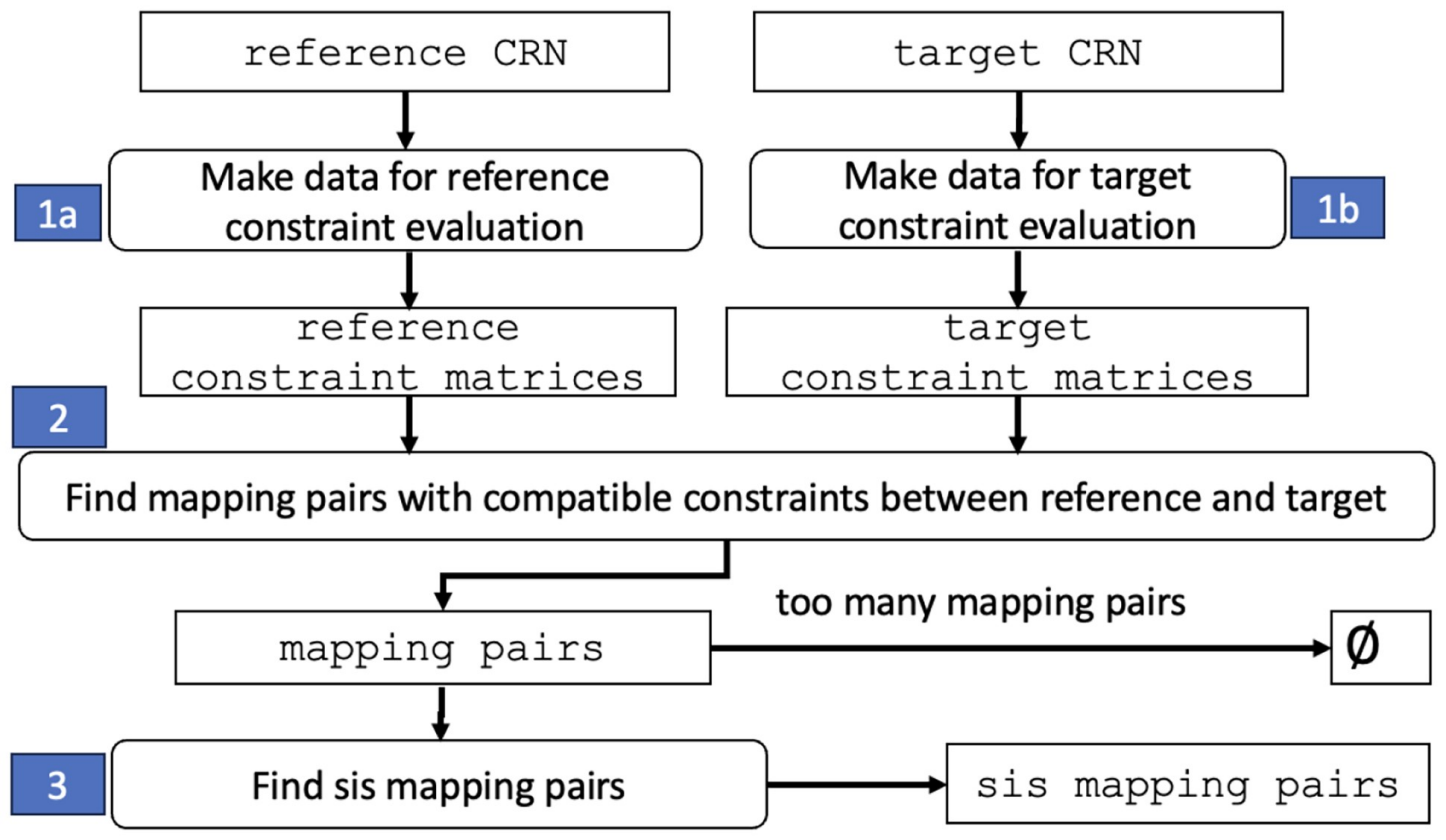
High-level algorithm for subnet discovery. Data are indicated by regular rectangles. Program logic is indicated by rounded rectangles and annotated with shaded numbers to indicate the sequence of steps.

Figure 4 displays our high-level algorithm for subnet discovery. Processing steps are indicated by rectangles with rounded edges, and data by rectangles with unrounded edges and text in a teletype font. Steps 1a and 1b construct constraints for the reference and target CRNs. Step 2 uses constraints to find a subset of mapping pairs that are potentially inferred networks in the target that are structurally identical to the reference. Step 3 evaluates mapping pairs to find sis mapping pairs.

The high-level program logic in Fig. 4 is quite similar to Yang *et al.* (2024). However, we proceed in a different way. First, we use constraints that are specific to the characteristics of CRNs, especially the nature of incoming and outgoing hyperarcs for reactions in CRNs. Second, we use matrix representations of constraints and the chemical network to achieve speedups through vectorization. Third, we provide a way to manage the “computational budget” by having a threshold on the number of mapping pairs that are evaluated. These points are addressed in detail in Section 3.

### 2.2 Generating random CRNs

Our choice of constraints and the way in which we evaluate our algorithms are based on a statistical characterization of CRNs in the BioModels repository (Xu 2023). The characterization focuses on reactions and the observation that a reaction can be classified by two numbers: its number of reactants (the sources for the reaction’s incoming arc) and its number of products (destinations for its outgoing arc). These two numbers constitute the **reaction type**. For example, a (1,2) reaction has one reactant species and two product species, such as A→B+C. Xu (2023) defines 16 reaction types, and describes the distribution of reaction types in BioModels. In pySubnetSB, the method NetworkBase.makeRandomNetworkByReactionType in the module network_base implements the random generation of CRNs according to this distribution.

The evaluation of subnet discovery requires generating reference–target pairs, where the reference is randomly embedded in the target. Our approach is implemented in the network_base method NetworkBase.RandomReferenceAndTarget. This method inputs the size of the reference CRN, $M_{s}^{R}$ species and $M_{r}^{R}$ reactions, and the size of the target CRN, $M_{s}^{T}$ species and $M_{r}^{T}$ reactions. The first step constructs a preliminary target CRN with $M_{r}^{T}-M_{r}^{R}$ reactions and $M_{s}^{T}$ species. Next, the reference CRN is generated with $M_{r}^{R}$ reactions and $M_{s}^{R}$ species using species that are randomly selected from the preliminary target CRN. Lastly, the target network is constructed by merging (and randomizing) the preliminary target network with a copy of the reference network.

## 3 Results

Here, we discuss the CRN-specific constraints that we employ (Section 3.1), their effectiveness in reducing computational complexity (Section 3.2), how we use vectorization and process-based parallelism and the speedups achieved (Section 3.3), and our approach to evaluating the statistical significance of subnet discovery (Section 3.4). Section 3.5 provides details of the package pySubnetSB.

### 3.1 Constraints for CRNs

pySubnetSB employs a constraint-based approach to reduce the number of mapping pairs. Constraints are custom-made to CRNs in two ways. First, different constraints are used in the construction of reaction mappings and species mappings (an approach that does not seem to be applied in other subgraph discovery algorithms). Second, many of our constraints are structured using the concept of reaction type discussed in Section 2.2, something specific to CRNs.

#### 3.1.1 Reaction constraints and reaction mappings

Let $r^{R}$ be a reaction in the reference CRN and $r^{T}$ be a reaction in the target CRN. A constraint is a predicate (i.e. a boolean-valued function) on $\left(r^{R},r^{T}\right)$ such that: (a) the result is always true if there is a sis mapping pair with $r^{R}$ mapped to $r^{T}$ and (b) the result is false as frequently as possible if there is no such sis mapping pair. Put differently, constraints should have no false negative, and constraints should minimize false positives (since false positive increase computation time).

Our first reaction constraint, RC1, is that $r^{T}$ and $r^{R}$ must be the same reaction type. For example, this constraint holds for $r^{R}=\mathrm{J5}$ in Model 1034 in Fig. 1b and $R^{T}=\mathrm{v01}$ because both reactions have two reactants and one product. Note that to simplify the tables, we encode the reaction type using two decimal digits so that (2,1) is represented as “21”.

The remaining constraints make use of the reaction monopartite graph, a graph in which all nodes are reactions. This graph is constructed from the full CRN bipartite hypergraph by placing an arc from a source reaction to a destination reaction if the source has a product species, i.e. a reactant for the destination. Figure 3 displays the reaction monopartite graph for Model 1034.

Reaction constraints RC2 and RC3 use information about predecessors and successors in the reaction monopartite graph. Consider the arc from reaction $J_{1}$ to $J_{5}$ in Fig. 3b. Here, $J_{1}$ is a 1-step predecessor to $J_{5}$, and $J_{5}$ is a 1-step successor to $J_{1}$. A 2-step predecessor is a predecessor of a 1-step predecessor, and similarly for a 2-step successor.

The reaction constraints are the following.

- RC1: $r^{R}$ and $r^{T}$ have the same reaction type.
- RC2: The count of 1-step and 2-step *predecessor* reactions (by type) for $r^{R}$ is no larger than the same count for $r^{T}$.
- RC3: The count of 1-step and 2-step *successor* reactions (by type) for $r^{R}$ is no larger than the same count for $r^{T}$.

**Figure 5.**
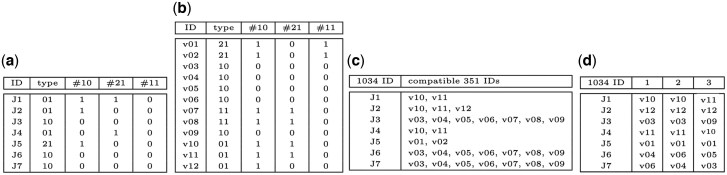
Illustration of calculating reaction mappings. (a and b) Partial data for reaction constraint matrices (reaction type, 1-step successor counts). (c) The reaction compatibility calculated from (a) and (b). (d) Three reaction mappings (the columns) of the many reaction mappings; the reaction mappings are based on (c) and are obtained by selecting a unique 351 reaction in the list of compatible reactions for each 1034 reaction.

Each CRN has a reaction and a species **constraint matrix** that are constructed in Steps 1a, 1b in Fig. 4. These matrices are used to evaluate constraints when constructing mapping pairs (Step 2 in Fig. 4). A row in the reaction constraint matrix corresponds to a reaction, and columns provide data used in constraint evaluations. There is a column for RC1 (reaction type). For RC2, there are two columns for each possible predecessor reaction type; cell values indicate the number of 1-step and 2-step predecessor reactions for the reaction type. Similarly, there are two columns for each reaction type for RC3. Figure 5a and b displays portions of the reaction constraint matrices for 1034 and 351, respectively. The columns RC2 and RC3 contain counts and are labeled with a hash (“#”) followed by a reaction type (encoded as a two-digit integer).

The construction of the reaction mappings involves building the **reaction compatibility vector**. The vector’s *i*th position is for the *i*th reference reaction and contains the set of reactions in the target that can be associated with the *i*th reference reaction (based on evaluating RC1–RC3). Figure 5c displays the reaction compatibility vector using the constraints in Fig. 5a and b. Figure 5d shows three of the many reaction mappings obtained from Fig. 5c by selecting a distinct 351 reaction in the list of compatible reactions for each 1034 reaction.

A reaction mapping is constructed by selecting one target reaction from each entry in the reaction compatibility vector without repeating a target reaction. Figure 5d illustrates three reaction mappings for the running example.

#### 3.1.2 Species constraints and species mappings

Species constraints are organized and implemented in a manner analogous to reaction constraints. There are species constraint matrices, species compatibility vectors, and species mappings obtained from species compatibility vectors.

Species are based on the full CRN bipartite graph. Let $s^{R}$ be the reference species and $s^{T}$ be the target species. The species constraints check that $s^{T}$ is referenced in reactions at least as often as $s^{R}$ is referenced.

- SC1: The count of reactions (by type) in which $s^{R}$ is a *reactant* does not exceed the corresponding count for $s^{T}$.
- SC2: The count of reactions (by type) in which the $s^{R}$ is a *product* does not exceed the corresponding count for $s^{T}$.
- SC3: The count of reactions (by type) that can be reached by $s^{R}$ within two steps in the *forward* direction does not exceed the same count for $s^{T}$.
- SC4: The count of reactions (by type) that can be reached by $s^{R}$ within two steps in the *reverse* direction does not exceed the same count for $s^{T}$.

### 3.2 Effectiveness of constraints

We quantify the effectiveness of reaction and species constraints by reducing reaction and species mappings. Our studies calculated the number of mappings for different combinations of constraints. For reactions, we considered the conditions: (i) no constraints (none), (ii) only RC1, (iii) only RC2 and RC3, and (iv) all constraints (RC1–RC3). For species, the conditions are: (i) no constraints, (ii) SC1–SC2, (iii) SC3–SC4, and (iv) SC1–SC4. Condition (i) (reactions and species) is calculated deterministically as in Eq. (1). The remaining conditions are evaluated using 1000 iterations of random networks. An iteration for reaction (species) constraints considers one of conditions (ii)–(iv). We use Section 2.2 to generate a random target CRN with 100 reactions and 100 species that has a subnet that is the reference with 20 reactions and 20 species. Then, we execute steps 1a, 1b, and 2 in Fig. 4 for reactions (species), and count the number of reaction (species) mappings using the method CompatibilityCollection.log10_num_assignment in the pySubnetSB module compatibility_collection.py.

**Figure 6.**
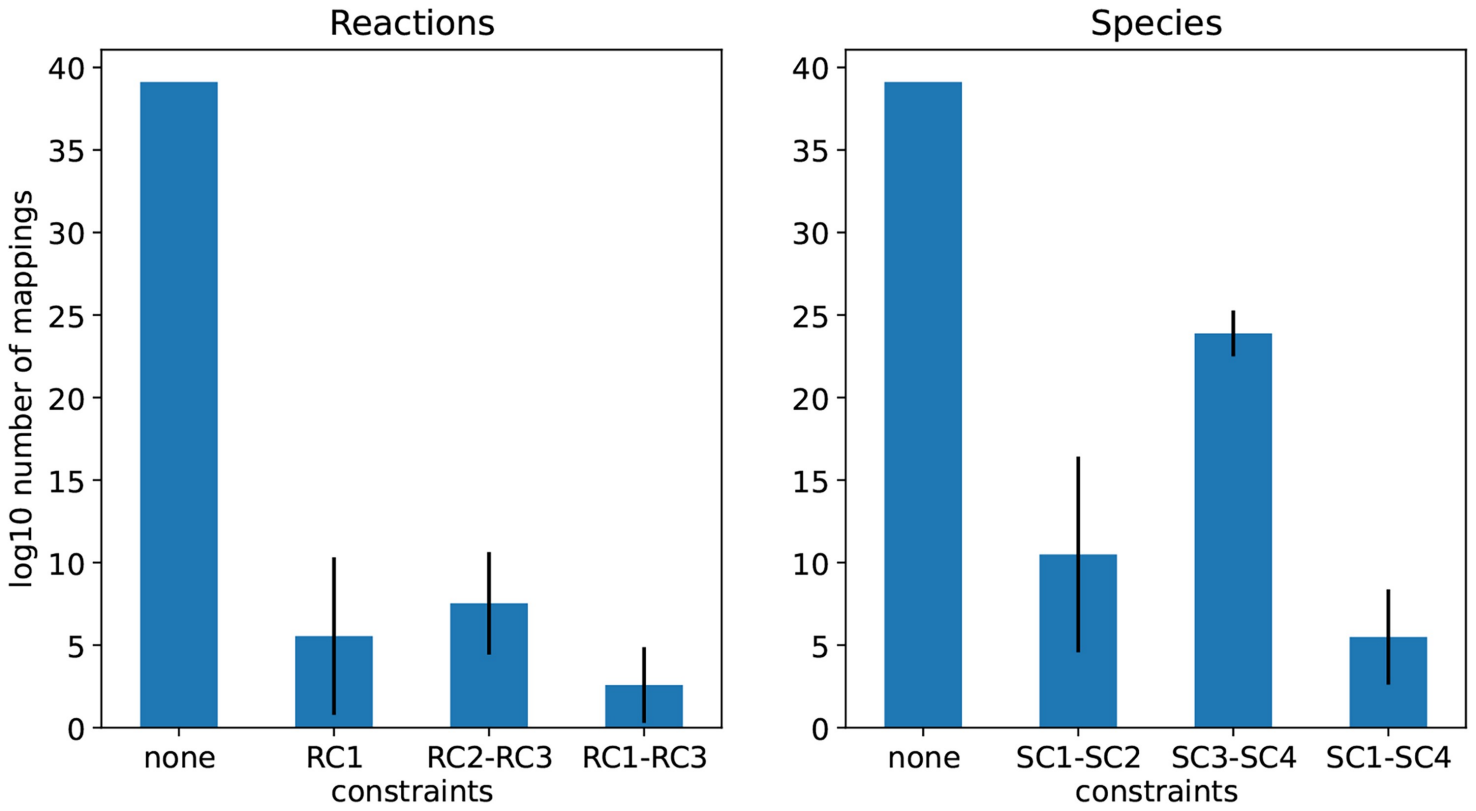
Constraints reduce the number of mappings to be evaluated by over $10^{30}$ for both species and reactions. The plots report the $log_{10}$ of the number of mappings to be evaluated for combinations of constraints. Solid bars are median values from simulation studies, and vertical lines indicate sample standard deviations.

Figure 6 displays a bar plot of the results of these studies. The vertical axis is the number of mappings in units $log_{10}$, and the horizontal axis indicates combinations of constraints (conditions). The vertical lines are standard deviations. If no constraint is applied, the number of mappings is very close to $10^{40}$ for both reactions and species, and so is almost $10^{80}$ mapping pairs (the product of the number of species and reaction mappings). Applying all constraints in combination, we have approximately $10^{3}$ mappings for reactions and $10^{5}$ mappings for species, or $10^{8}$ mapping pairs. This is a reduction by a factor of more than $10^{70}$ in the number of mapping pairs compared to the number that does not have constraints.

**Figure 7.**
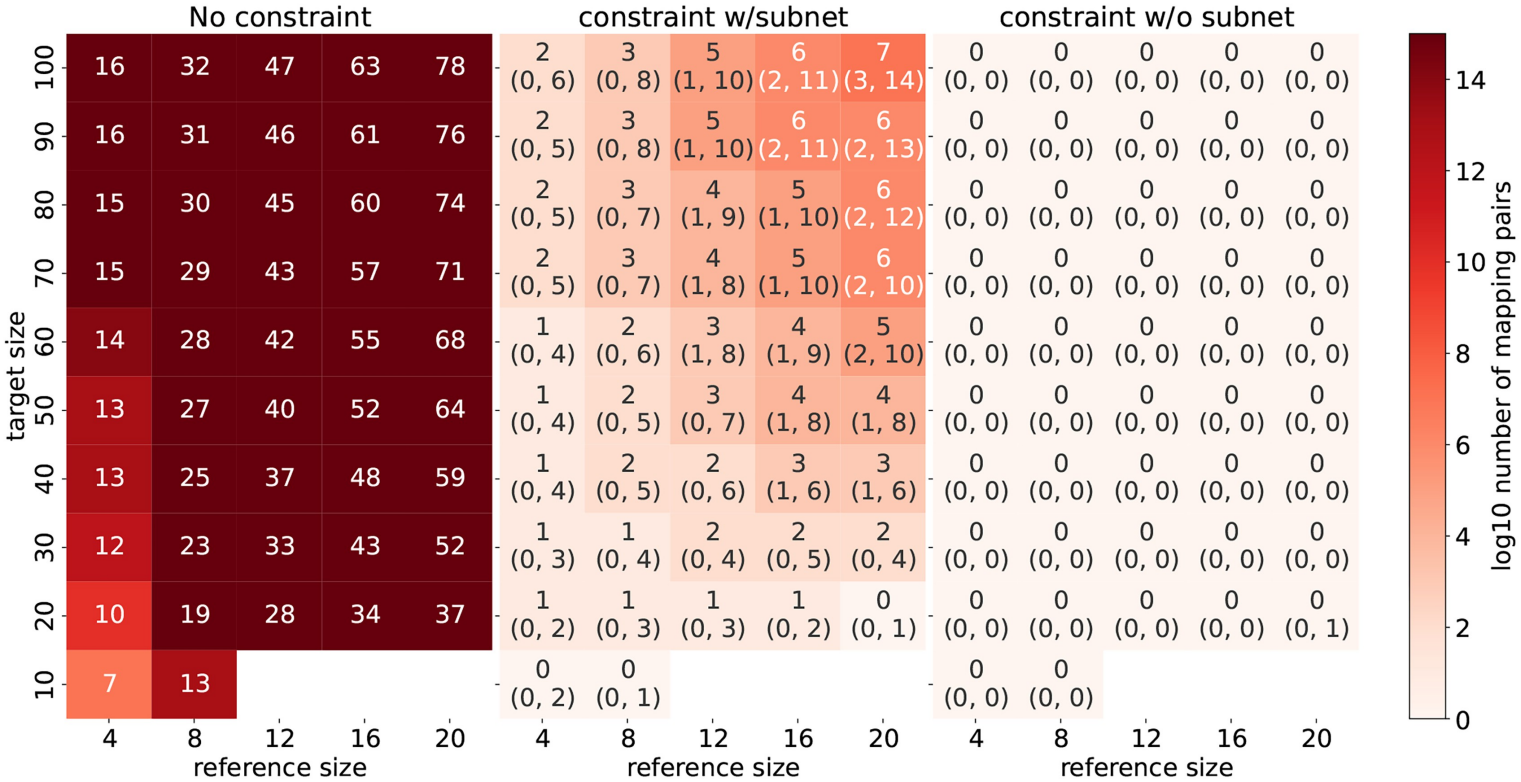
Constraints reduce the number of mapping pairs to be evaluated when a reference network is present and even more when it is absent. Unparenthesized numbers are the integer part $log_{10}$ of the median number of mappings of 1000 simulations; parenthesized numbers are the 10% and 90% percentiles of 1000 simulations.

Figure 7 uses the same procedure as above to study the number of mapping pairs under three conditions: (a) no constraints; (b) all constraints when the reference is present in the target; and (c) all constraints when the reference is not present in the target. Cells are annotated with the integer part of $log_{10}$ of the number of mapping pairs obtained from the simulation; numbers in parentheses are the 10th and 90th percentiles obtained from simulation. (Parenthesized numbers are not needed for “no constraint” since this is a deterministic calculation.) The reference size is the horizontal axis; the target size is the vertical axis; darker colors indicate a larger number of mapping pairs. We see that constraints provide a huge reduction in mapping pairs, even for larger references and targets. Also of note is the effectiveness of constraints in detecting the *absence* of the reference in the target. We see that for this condition and with very large CRNs, the number of mapping pairs is quite small.

### 3.3 Computational speedups

Constraints dramatically reduce the number of mapping pairs. However, even after constraints are applied, there may still be $10^{10}$ to $10^{13}$ mapping pairs. The evaluation of these many mapping pairs requires considerable computation.

pySubnetSB achieves computational speedups in two ways. The first approach is **vectorization**, the use of matrix operations instead of executing for loops. pySubnetSB uses vectorization in the construction of mapping pairs that comply with constraints (step 2 in Fig. 4) and in the evaluation of mapping pairs (step 3 in Fig. 4). In particular, the latter requires testing for equality between the reference stoichiometry matrix and the permuted subset of the target stoichiometry matrix specified by the mapping pair. The foregoing can be done for one mapping pair at a time. pySubnetSB implements a scheme for stacking stoichiometry matrices so that *m* mappings can be evaluated simultaneously.

We conducted studies measuring the runtime for stacking *m* matrix comparisons in the evaluation of mapping pairs; m∈{1,10,100,1000}. The studies used randomly generated reference networks (five reactions, five species) that were embedded in randomly generated target networks (100 reactions, 100 species) as described in Section 2.2. Subnet discovery was done on one core of a 128 GB Mac Studio M1 with 20 cores. Let $T_{M}(m)$ be the execution time for stacking *m* stoichiometry matrices. The speedup is $S_{M}(m)=\frac{T_{M}(1)}{T_{M}(m)}$. The results indicated a substantial speedup from the use of vectorization: $S_{M}(10)\approx 9,S_{M}(100)\approx 30,S_{M}(1000)\approx 40$. No significant improvement was observed for m>1000.

pySubnetSB also implements process parallelism using the Python multiprocessing package to take advantage of systems with multiple processors (cores). As a result, step 3 in Fig. 4 evaluates the mapping pairs in parallel on many processors.

**Figure 8.**
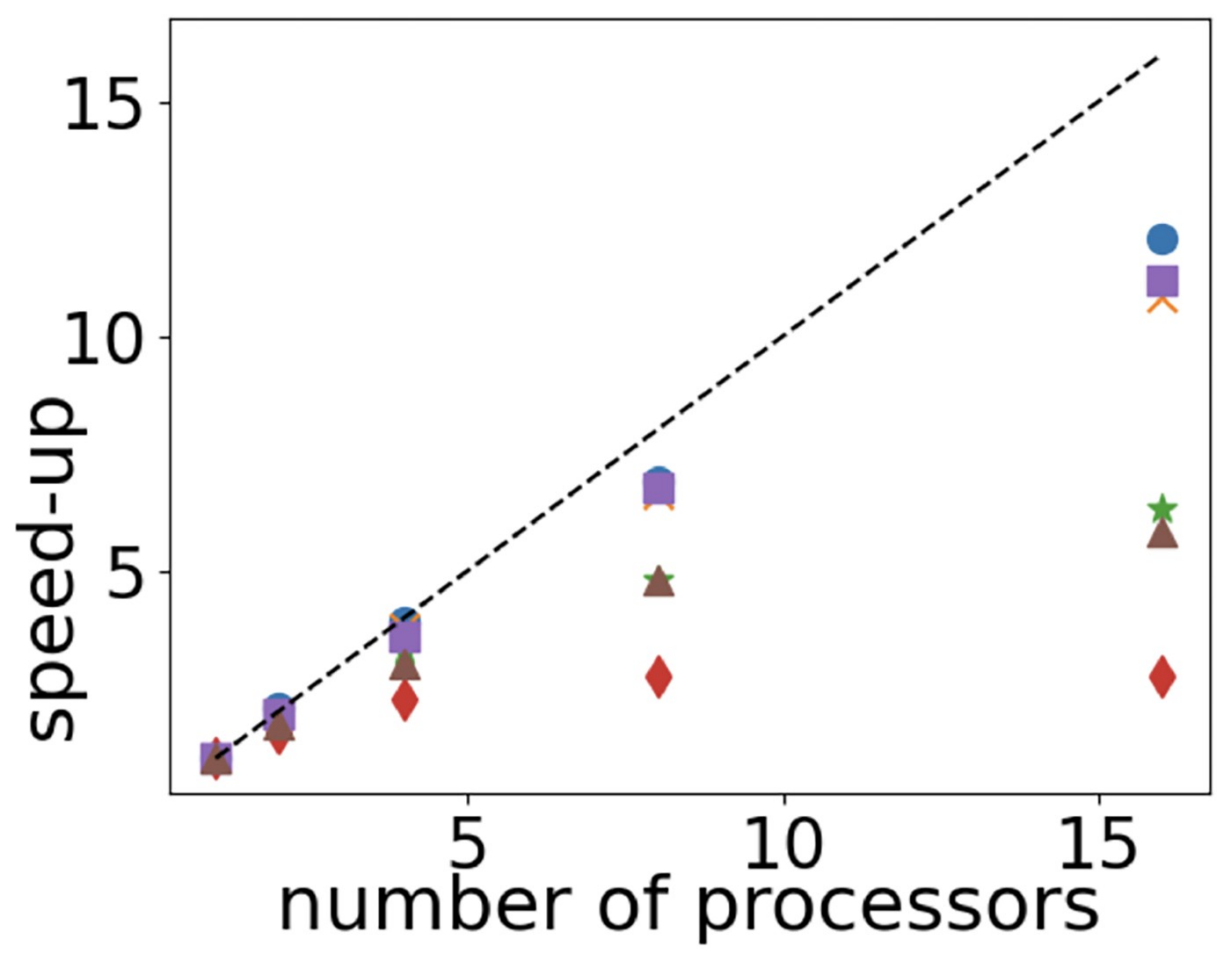
Speedup from process parallelism. Markers indicate studies. Speedups are larger when there are more mapping pairs.

To assess the speedup for process parallelism, we conducted six studies with a different set of 10 random target CRNs (100 reactions, 100 species); each target CRN had an embedded reference CRN (five reactions, five species). These networks were generated using the method in Section 2.2. Then, subnet discovery was done using 1, 2, 4, 8, and 16 processors on a 128 GB Mac Studio M1 (with 20 cores). Let $T_{P}(n)$ be the time for subnet discovery on *n* processors. The study calculated the speedup as $S_{P}(n)=\frac{T_{P}(1)}{T_{P}(n)}$. Figure 8 reports the results. Marker shapes indicate speedups for different studies. The dashed line is the ideal speedup, which is $S_{P}(n)=n$ or linear speedup. We see that a near linear speed up is achieved in three of the studies (although less than linear for 16 processors). As expected, a near linear speed is achieved for studies with a large number of mapping pairs since there is more parallel work.

Computational speedups are multiplicative. So, a speedup of 40 from vectorization and a speedup of 10 from process parallelism results in an overall speedup of 400. That is, a subnet discovery that would take 1 day on a single processor evaluating one mapping pair at a time will instead have an elapsed of 3.5 min on 15 processors that use vectorization of 1000 mapping pairs.

### 3.4 Statistical significance of subnet discovery

Some CRNs are quite small. Since there are many more instances of small CRNs than larger ones, it is possible that the subnet discovery for a small CRN is not statistically significant.

We assess statistical significance by answering the question “How likely is the reference CRN to occur by chance?” This depends on the number of reactions and species in the reference, which are denoted by $\left(M_{s}^{R},M_{r}^{R}\right)$. We calculate the probability of “occurring by chance” (null distribution) using randomly generated CRNs as described in Section 2.2.

- Step (1): generate $K^{R}$ reference CRNs with size $\left(M_{s}^{R},M_{r}^{R}\right)$;
- Step (2): generate $K^{T}$ target CRNs also of size $\left(M_{s}^{R},M_{r}^{R}\right)$;
- Step (3): for each reference CRN in step (1), count the number of target CRNs in step (2) that are strongly structurally identical, and report the fraction of occurrences of strong structural identity.

$K^{R}$ , $K^{R}$ should be sufficiently large so that there is little variability for the statistics calculated in step (3). We find that it is sufficient to use $K^{R}=100,K^{T}=1000$.

**Figure 9.**
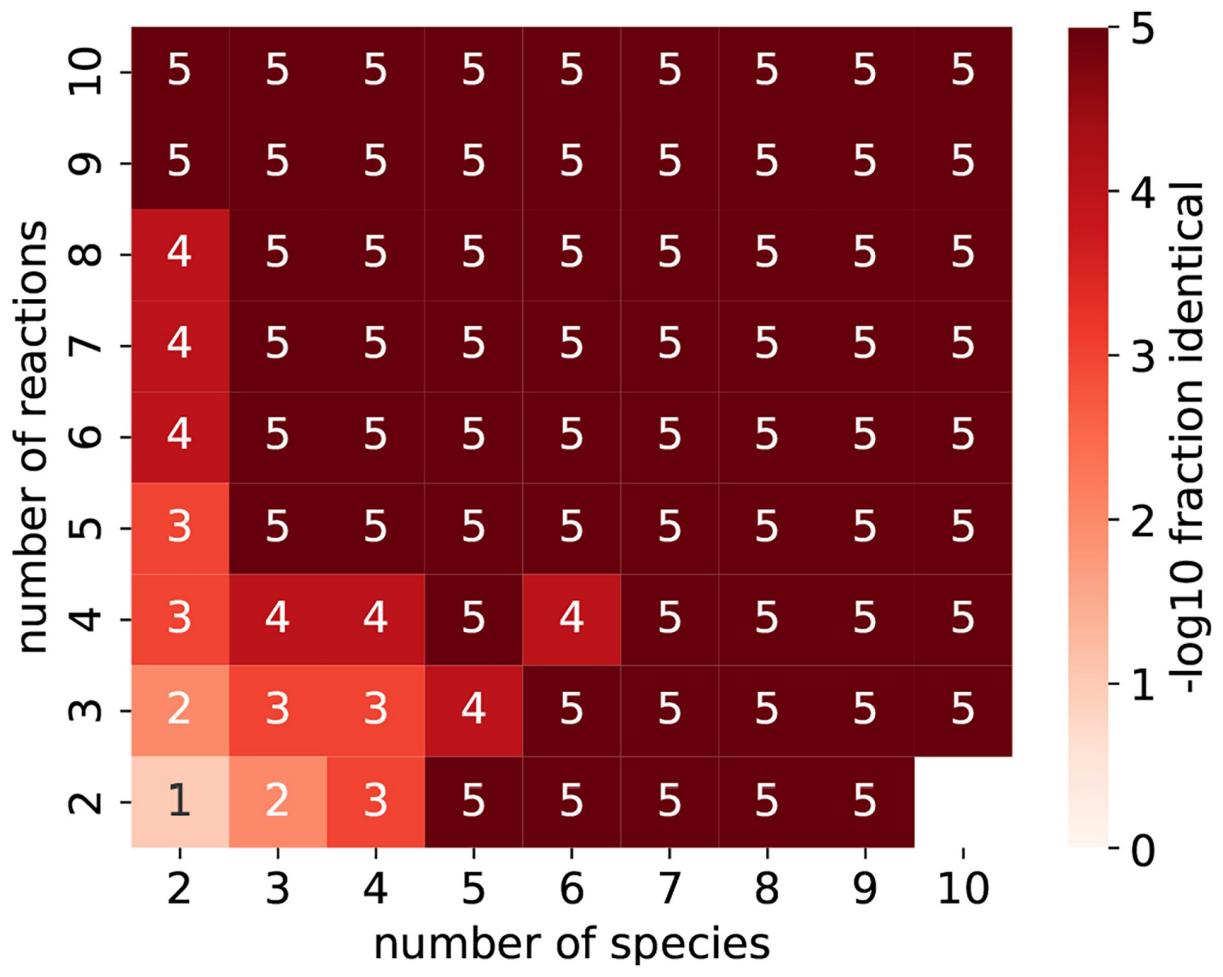
Statistical significance of randomly chosen CRNs. Cells are annotated with −logp, where *p* is the probability of a randomly chosen CRN as described in Section 3.4. The horizontal axis is the number of species, and the vertical axis is the number of reactions.

Figure 9 displays the result of the above approach for network sizes from (2,2) to (10,10). The horizontal axis is the number of species in the model, and the vertical axis is the number of reactions. Cells are annotated with the integer part of $-log_{10}$ of the fraction of random CRNs that are strongly identical under the null distribution (i.e. the Type 1 error in statistical inference (Lindgren 1993)). For example, an annotation of “1” means a significance level of 0.1. (A “5” means that the step found either 1 or 0 structurally identical networks.) In the sequel, we use Fig. 9 to evaluate the statistical significance.

### 3.5 Python package

pySubnetSB is an open source Python package. Models can be represented in the SBML (XML) or in the human-readable Antimony model description language (Smith *et al.* 2009). The package is installed using pip install pySubnetSB.

The basic API consists of two functions. The first is findReferenceInTarget that takes three arguments: reference_model, target_model, and max_num_mapping_pair. The reference model is the first argument, and the target model is the second argument. The function returns an indicator of whether subnet discovery was aborted because the number of mapping pairs exceeded max_num_mapping_pair. The second API is findReferencesInTargets, which generalizes the first API to do subnet discovery for directories of models. More details can be found in the Jupyter notebook api_basics.ipynb in https://github.com/ModelEngineering/pySubnetSB/blob/main/examples/.

## 4 Discussion

**Figure 10.**
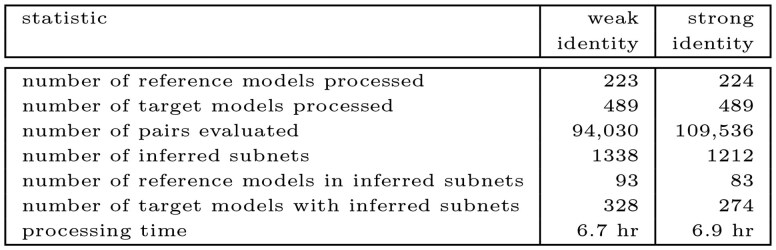
Summary of subnet discovery for curated BioModels.

This section studies the occurrence of subnets in the curated branch of BioModels, approximately 1000 models. (See data/README.md in the github repository for details.) Reference CRNs are models with no more than 10 reactions; target models have more than 10 reactions. To manage computational demands, we set the API parameter max_num_mapping_pair so that we evaluate at most $10^{12}$ mapping pairs for a subnet discovery. Both weak and strong identity are considered. Figure 10 summarizes our studies for approximately 200 000 reference-target pairs. The elapsed time for the studies was slightly less than 14 h on a 128 GB, 20 core Mac Studio M1.

### 4.1 Use Case 1: inferring function in targets

Here, we consider Use Case 1 in the introduction, that the presence of the reference CRN in the target suggests that functions of the reference are in the target. The reference CRN is Model 10, which studies ultrasensitivity and negative feedback in a mitogen-activated protein kinase (MAPK) cascade. The model has 10 reactions and seven species. It turns out that this reference CRN is a subnet of several target models: (i) Model 146 (34 reactions), which investigates MAPK and Akt pathways in heregulin-induced ErbB signaling; (ii) Model 270 (42 reactions), which analyses isoform-specific ERK signaling and cell fate decisions; and (iii–iv) Models 466 (28 reactions) and 468 (74 reactions), which study shear-stress-induced nitric oxide production in endothelial cells. We have strong confirmation that the MAPK function is present in the targets since the paper associated with each model explicitly states that MAPK function is part of their model.

**Figure 11.**
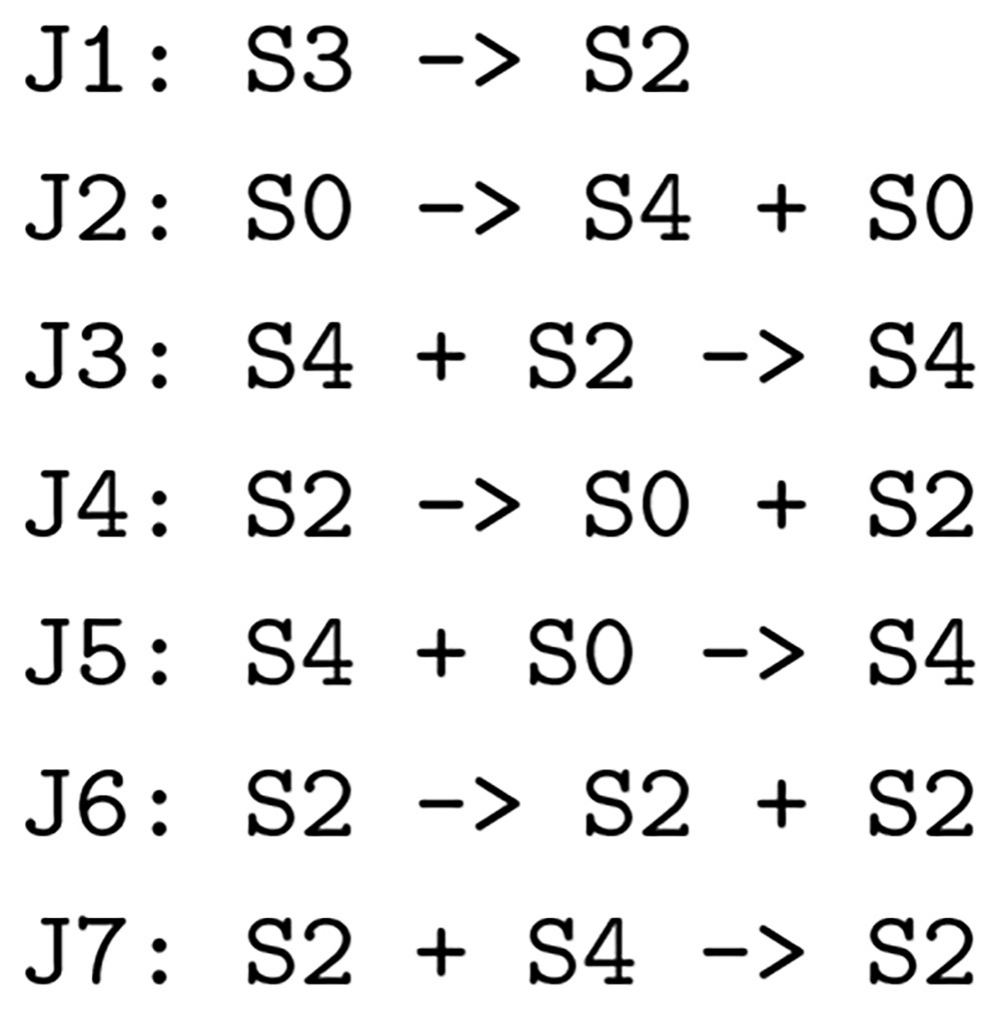
An oscillating CRN, i.e. a subnet of several models in BioModels.

We present another example of Use Case 1. This example lacks the strong confirmation provided in the first example, but it illustrate how subnet discovery can be used to generate interesting hypotheses. We ask the question “Are there models in BioModels that contain hidden oscillators?” We are aided in pursuing this question by the existence of a substantial database of generated oscillatory CRNs constructed as described in Tatka *et al.* (2023). We used 6000 of these generated models as reference CRNs and all of BioModels as target CRNs to do subnet discovery with weak identity. It turns out that one of the reference models, the oscillator in Fig. 11, is a statistically significant subnet of three models in BioModels: (i) Model 480 (mucosal immune responses during *Helicobacter pylori* infection), (ii) Model 695 (effect of blue light irradiation on reducing the severity of psoriasis vulgaris), and (iii) Model 872 (HIV-associated immunosuppression on HPV persistence in the oral mucosa). The published papers for the targets make no reference to oscillatory characteristics in these models.

### 4.2 Use Case 2: inferring common mechanisms

A common mechanism seems plausible if: (a) there are multiple targets that have an inferred network for the same reference; and (b) there is plausible explanation for why the targets might embed the same reference CRN. A potential common mechanism in our studies is Model 1059, which addresses the regulation of caspase-3 activation and degradation in apoptosis and has seven reactions and three species. The target models in BioModels that have an inferred subnet for 1059 are: Model 156 (oscillations and variability in the p53 system); Model 546 (early immune response and adaptive immune response kinetics in mice infected with influenza A virus); Model 789 (interaction between cancer cells and an oncolytic virus); Model 1012 (cell therapy in B-cell acute lymphoblastic leukemia); and Model 1048 (designing a cancer therapy). We note that all targets are related to intracellular immune response and/or cell death, and apoptosis is key to these processes. This gives us an interesting hypothesis about a common mechanism.

## 5 Conclusions and future work

Subnet discovery is a powerful tool for generating hypotheses related to the presence of function and conserved mechanisms in CRNs. Subnet discovery is a kind of subgraph problem, and these problems are extremely demanding computationally.

This article introduces pySubnetSB, an open source Python package that discovers subnets in CRNs represented using the SBML community standard. pySubnetSB employs a constraint-based approach that greatly reduces computations. In our studies of randomly selected target networks with 100 reactions that each embed a distinct reference networks with 20 reactions, the number of mapping pairs is reduced from a computationally infeasible $10^{78}$ to a more practical $10^{8}$. pySubnetSB employs a constraint-based approach that greatly reduces computations (e.g. in our studies, from the evaluation of an infeasible $10^{78}$ mapping pairs to a more practical $10^{8}$ mapping pairs). Furthermore, our implementation provides large speedups as a result of vectorization and process-based parallelism (e.g. a speedup of 400 in our studies). In addition, we develop a methodology that assesses the statistical significance of subnet discovery. Last, we use pySubnetSB to study subnets in BioModels for approximately 200 000 pairs of reference and target models. We show that for a reference MAPK pathway, subnet discovery correctly indicates the presence of MAPK function in several target models. The studies also suggest a couple of interesting hypotheses: (a) the presence of hidden oscillators in several models in BioModels, and (b) a conserved mechanism for intracellular immune response.

In the near term, we want to increase the computational speed of pySubnetSB by making use of Graphics Processing Units (GPUs). Longer term, we plan to explore the hypotheses identified in our discovery of subnets in BioModels.
